# Superhost Plants Alter the Structure of Plant–Galling Insect Networks in Neotropical Savannas

**DOI:** 10.3390/plants8100369

**Published:** 2019-09-24

**Authors:** Walter Santos de Araújo, Leuzeny Teixeira Moreira, Luiz Alberto Dolabela Falcão, Magno Augusto Zazá Borges, Marcílio Fagundes, Maurício Lopes de Faria, Frederico Augusto Guimarães Guilherme

**Affiliations:** 1 Departamento de Biologia Geral, Universidade Estadual de Montes Claros, Montes Claros, MG 39401-089, Brazil; walterbioaraujo@gmail.com (W.S.d.A.); luizdolabelafalcao@gmail.com (L.A.D.F.); diptera@gmail.com (M.A.Z.B.); marcilio.fagundes@gmail.com (M.F.); fariaml@gmail.com (M.L.d.F.); 2 Programa de Pós-Graduação em Biodiversidade e Uso dos Recursos Naturais, Universidade Estadual de Montes Claros, Montes Claros, MG 39401-089, Brazil; 3 Instituto de Biociências, Universidade Federal de Goiás, Regional Jataí, Jataí, GO 75804-020, Brazil

**Keywords:** ecological networks, galling insects, host specificity, plant–insect interactions, *Qualea*

## Abstract

Host plants may harbor a variable number of galling insect species, with some species being able to harbor a high diversity of these insects, being therefore called superhost plants. In the present study, we tested the hypothesis that the occurrence of superhost plant species of genus *Qualea* (Vochysiaceae) affects the structure of plant–galling insect ecological networks in Brazilian Cerrado. We sampled a total of 1882 plants grouped in 131 species and 43 families, of which 64 species and 31 families of host plants hosted 112 galling insect species. Our results showed that occurrence of superhosts of genus *Qualea* increased the linkage density of plant species, number of observed interactions, and the size of plant–galling insect networks and negatively affected the network connectance (but had no effect on the residual connectance). Although the occurrence of *Qualea* species did not affect the plant species richness, these superhosts increased the species richness and the number of interactions of galling insects. Our study represents a step forward in relation to previous studies that investigated the effects of plant diversity on the plant–insect networks, showing that few superhost plant species alter the structure of plant–herbivore networks, even without having a significant effect on plant diversity.

## 1. Introduction

The diversity of host plants is one of the main factors influencing the distribution of herbivorous insects in tropical environments [1,2,3,4]. Many herbivorous insects use the host plants, not only for their feeding, but also for their nesting and development, as is the case of endophagous insects. Endophagous insects are herbivores that develop part of their life cycle inside plant tissues [5], as is the case of flower-head insects, fruit-flies, insect miners, and galling insects [3]. This latter group, also called cecidogenous insects, is known to contain the most sophisticated herbivores of nature [6], since they are the only insects capable of modifying the physiological and anatomical structure of host plants and induce the formation of galls [7]. Galls are structures formed by hyperplasia and hypertrophy of plant tissues [8], inside which the inductor insects (i.e., larvae or nymphs in developing) feed, complete their development, and still obtain shelter against natural enemies and environmental stressors [7]. Due to the high degree of intimacy of the galling insects and its host interactions, most species of galling insects tend to be highly specialized in their host plants [3,9,10,11]. On the other hand, host plants of galling insects may harbor a variable number of galling insect species [12], with some species being able to harbor a high diversity of these insects, being therefore called superhost plants [12,13,14].

Evidence suggests that superhost plants may influence the diversity of galling insects between different environments [12,13,14,15,16]. The richness of galling insects in African savannas is strongly influenced by the presence of superhost Terminalia sericea (Combretaceae) [13]. Similar results were obtained in Neotropical savannas where the presence of superhost *Qualea parviflora* (Vochysiaceae) substantially increased the richness of galling insects [12]. In the literature are documented several genera and species of superhost plants, such as *Baccharis* [17], *Copaifera* [16,18], *Eucalyptus* [19], and *Quercus* [20], although the effect of the presence of these taxa on the local diversity of galling insects has not been investigated for most cases. As the presence of superhost species tends to influence the richness of galling insects, it is also expected that these hosts potentially influence the diversity of interactions and the structure of ecological networks formed by plants and galling insects.

Ecological networks are sets of species (i.e., nodes) connected through ecological interactions (i.e., links) [21], where it is possible to quantify by different ecological indexes the distribution and density of the interactions [22]. The connectivity of the interactions within the network can be used as an indication of community specialization, since highly connected networks tend to have a low specialization [23]. Although there are few networks involving galling arthropods and host plants with the structure described in the literature, e.g., [11,24], there is evidence that these networks are weakly connected and have highly compartmentalized interactions. This is due to the species of galling insects consuming one or a few species of phylogenetically related plant species [9,11,24], so that each species of galling insect tends to develop only one link within the network. On the other hand, because plants vary greatly in the number of links that can contribute to network connectivity, superhost plants tend to add many interactions. Thus, the presence and diversity of superhost plant species is expected to increase the connectivity of plant–galling insect ecological networks.

**Figure 1:**
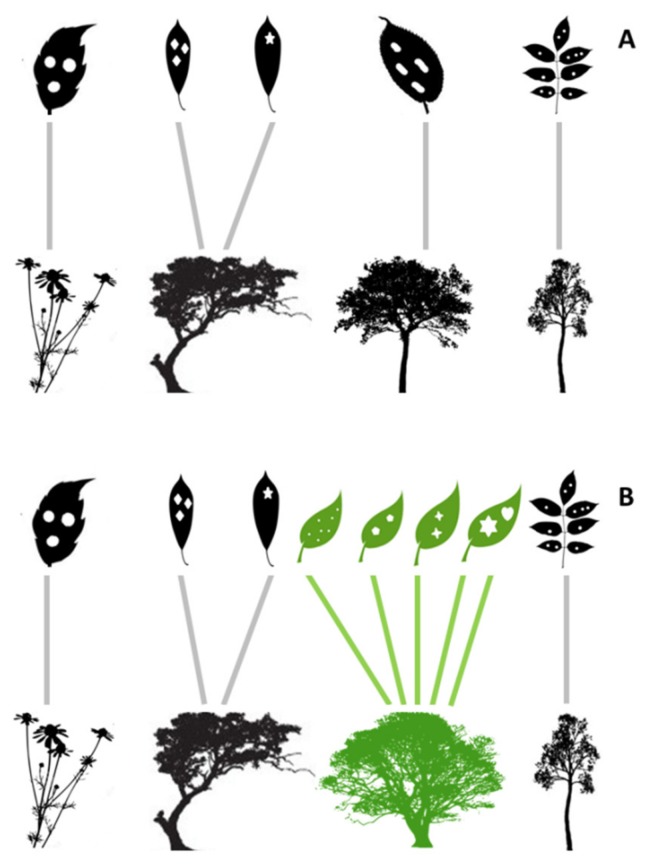
Ecological networks including interaction chains between galling insects (illustrated as insect gall morphotypes in the plant leaves) and host plants. (**A**) A network composed by host plant species (black plants) and galling insect species (black leaves with white insect galls) tends to be characterized by a low number of interactions. (**B**) Occurrence of a superhost plant species (green plant) can affect the number of galling species (green leaves with white insect galls) and links (green arrows) in the network and consequently alters the diversity and connectivity of the plant–galling insect network.

In the present study, we investigated the effect of superhost plant species on the structure of ecological networks composed by galling insects and host plants in areas of Neotropical savannas in Brazil. Neotropical savannas have a huge diversity of vegetation types [25,26], host plants [12], and galling insects [12,27]. We described the structure of plant–galling insect networks using linkage density of plants, number of observed interactions, network size, and network connectance [22]. The linkage density of plants and number of observed interactions were used as measures of diversity of interactions at plant and network level, respectively. On the other hand, network size is a measure of species diversity in the network, and network connectance is an inverse measure of network specialization [24]. Because superhost plant species tend to add many links in networks, we hypothesized that networks with higher occurrences of superhosts will have more realized links, larger network size, and greater network connectance (i.e., low specialization) (Figure 1).

## 2. Results

In total, we sampled 1882 plants grouped in 131 species and 43 families, of which 64 species and 31 families of host plants hosted 112 galling insect species (Table S1). The greatest number of gall morphotypes recorded occurred in leaves (83.9%), compared with stems (13.4%) and other organs (2.7%). Vochysiaceae was the family that hosted the greatest galling richness with 19 species, and *Qualea* was the most important host genus with 18 gall-inducing insect species recorded. The species *Qualea parviflora*, *Qualea multiflora*, and *Qualea grandiflora* hosted eight, seven, and three gall morphospecies, respectively. The species of *Qualea* had 6 ± 2.6 (mean ± SD) galling morphotypes, while the other species had mean of 1.5 (± 0.9). The occurrence of *Qualea* species did not influence the plant species richness (*R*^2^ = 0.188, *p* > 0.05), but the linkage density of plant species was significantly higher for plant–galling insect networks with the presence of *Qualea* than in the areas with no superhosts (Table 1).

**Figure 2:**
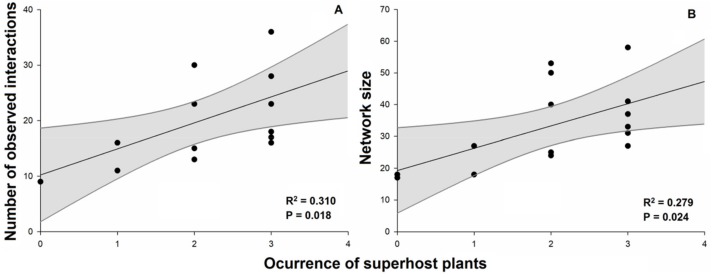
Effects of occurrence of superhost plants on the (**A**) number of observed interactions and (**B**) network size of plant–galling insect networks in Brazilian Cerrado. Occurrence of superhost plants is an ordinal variable of occurrence of *Qualea* species (number of species) to each network. Black points represent different plant–galling insect networks. Gray bands represent the model’s 95% confidence intervals.

**Figure 3:**
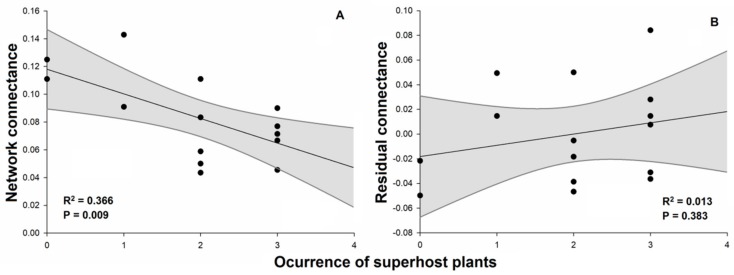
Effects of occurrence of superhost plants on the network connectance (**A**) and effect of network size on the connectance (**B**) of plant–galling insect networks in Brazilian Cerrado. Occurrence of superhost plants is an ordinal variable of occurrence of *Qualea* species (number of species) to each network. Black points represent different plant–galling insect networks. Gray bands represent the model’s 95% confidence intervals.

**Figure 4:**
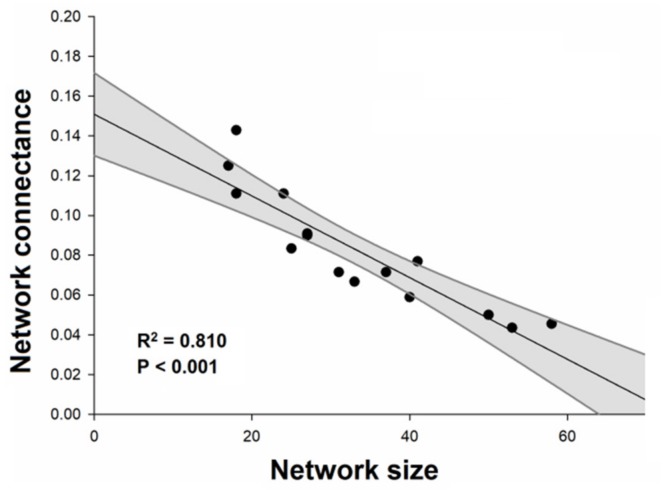
Effects of network size on the network connectance of plant–galling insect networks in Brazilian Cerrado. Occurrence of superhost plants is an ordinal variable of occurrence of *Qualea* species (number of species) to each network. Black points represent different plant–galling insect networks. Gray bands represent the model’s 95% confidence intervals.

Plant–galling insect networks ranged from 9 to 36 (19.6 ± 8.4) interactions. We found a positive effect of occurrence of superhost plants on the number of observed interactions (Figure 2A; Table 1). The number of species in the networks (network size) ranged from 18 to 58 (33.3 ± 13.0) species and also was positively influenced by the occurrence of superhost plants (Figure 2B). Values of network connectance in general were low, ranging from 4.3% to 14.3% (8.2% ± 2.9%). The connectance was negatively influenced by the occurrence of superhost plants (Figure 3A), but no effect was observed for the residual connectance (Figure 3B). The analyses also showed a negative relationship between network size and network connectance (Figure 4).

## 3. Discussion

Our findings suggested that superhost plants significantly alter the structure of plant–galling insect networks in Neotropical savannas. Corroborating our expectations, the occurrence of superhosts of genus *Qualea* increased the number of observed interactions and the size of plant–galling insect networks. On the other hand, our hypothesis that the occurrence of superhosts would increase the network connectance was not corroborated—on the contrary, we found a negative effect of the occurrence of superhosts on network connectance and no effect on the residual connectance. Although previous studies have investigated the effect of superhost taxa on the diversity of galling insects (e.g., [12,13]), this is the first study that addresses the importance of these host plants for the structure of plant–galling insect networks.

Plant species of genus *Qualea* were very important hosts for galling insects and drivers for network structure in our study. With only three plant species (*Qualea grandiflora*, *Qualea multiflora* and *Qualea parviflora*), which correspond to 2% of the 131 plant species recorded, the genus *Qualea* hosted 16% of the total number of galling species (18 morphospecies). Evidences points that genus *Qualea* is composed by species abundant and widely distributed in the Neotropical savannas [4,28,29,30], which was corroborated in the present study where *Qualea* occurred in 13 of the 15 sampled areas. According to Araújo et al. [12], the wide distribution of *Qualea* species leads to a high local number of galling insects morphospecies registered in each area (i.e., alpha diversity) and the large turnover of morphospecies among different localities (i.e., beta diversity). As a consequence, *Qualea* species contribute greatly to increasing the diversity of galling insect species within the networks, positively affecting the size of the networks and the diversity of realized interactions. It is important to note that there was no significant effect of the occurrence of *Qualea* species on plant species richness, so the effects of these superhosts on the network size and number of observed interactions are due to increase in species richness and interactions of galling insects, respectively.

Hypothetically network connectance is expected to range from 0% (no interactions) to 100% (perfectly connected). In trophic networks, connectance tends to be low (i.e., less than 50%), although it may vary depending on the type of ecological interaction [24,31,32]. For example, endophagous herbivore networks tend to be less connected than exophagous herbivore networks [24]. The values of connectance obtained in our study (mean of 8.2%) are higher than previously recorded for other plant–galling arthropod networks [11,31]. For example, Araújo et al. [11] recorded connectance of 2.7% in a network composed by galling insects and woody plants in the city of Nitra, Slovakia. In the same locality, Araújo and Kóllar [31] recorded a connectance of 4.9% for a network composed by galling mites and host plants. These differences can be due to the distinct sizes of these networks, because in the network of Araújo et al. [11] were analyzed 144 species (90 galling species and 54 host plant species), whereas in the network of Araújo and Kóllar [31] were analyzed 55 species (31 eriophyoid species and 24 host plant species). In our study, the largest network had 58 species (36 galling species and 22 host plant species). Previous studies indicate a negative correlation between the network size and connectance [24,32], which may affect the comparison between different ecological networks [24,33,34]. This is because networks with a high degree (i.e., with many species) tend to have a very high potential number of interactions [32]. In this sense, very large networks tend to have low connectivity (see Figure 4), because regardless of the number of interactions they perform, they always have a very large number of possible interactions.

When we evaluated the effect of the occurrence of superhosts on the network connectance, we found a negative correlation between variables. As previously commented, galling insects usually interact with one or a few plant species, while host plants can form connections with multiple species of galling insects. This feature is especially striking in superhost plants. Thus, the presence of a superhost plant increases the network size much faster than the number of interactions in the network, since the galling species of these superhost plants are unable to interact with other plant species. In this sense, the expected increase in the realized interactions due to the presence of a superhost is offset by the increment in the network size that the superhost induces, resulting in a negative effect of occurrence of superhosts on network connectance. On the other hand, when we used residual connectance (to control the mathematical effect of network size on the connectance) [24], this effect disappeared. These findings indicate that the negative effect of superhosts on network connectance is due to the effect these plants have on the size of the network (see Figure 3B). Additionally, with the residual connectance, there is a tendency of a positive effect of the occurrence of superhosts, although the effect was not significant. This inversion in the data trends is due to the fact that in most of the areas (66%) where the three superhost species (*Qualea grandiflora*, *Qualea multiflora* and *Qualea parviflora*) occurred, the residual connectance was higher than expected by network size (positive residuals, i.e., >0). On the other hand, the two networks that did not have superhost species registered were less connected than expected by size (residual negatives, i.e., <0).

## 4. Materials and Methods

### 4.1. Study Area

**Figure 5:**
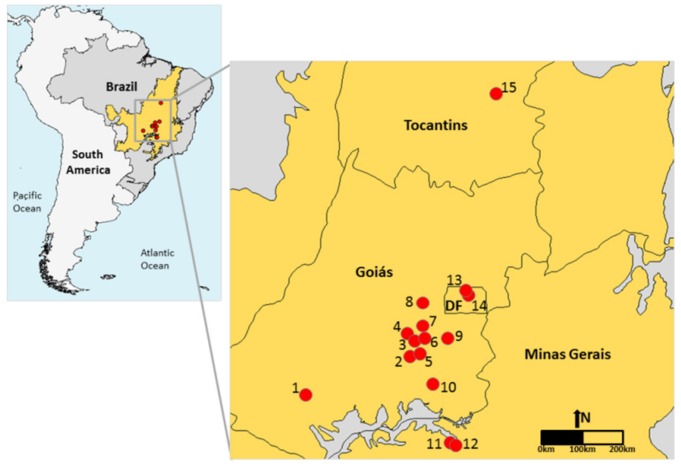
Location of the 15 areas of Neotropical savanna (red points) sampled in the Cerrado biome (orange area) in Brazil. Codes correspond to the areas indicated in Table 2.

The study was performed in 15 areas under the domain of Brazilian Cerrado in the states of Tocantins, Minas Gerais, Goiás, and Federal District (Table 2; Figure 5). These regions are characterized by a climate classified as Aw by Köppen (tropical rain) with occurrence of dry winters and rainy summers. The region has an average annual rainfall of about 1500 mm, ranging from 750 to 2000 mm [25]. The vegetation studied is classified as Neotropical savanna (i.e., cerrado *sensu stricto*), which is dominated by sclerophyllous plants that occur in poor soils, where the availability of water and soil mineral nutrients is limited [26].

### 4.2. Sampling of Plant–Galling Insect Interactions

The sampling was performed in 150 plots of 10 x 10 m randomly distributed in the study areas (10 plots per area). We used this design to standardize sampling between study areas. In each plot, all woody plants with a circumference greater than 15 cm at ground level were sampled. Each plant sampled was cataloged and identified in the field, and leaves, stems, and flowers were inspected in the search for galls. Galling insects were sampled in the field and classified as galling insect morphospecies according to morphological characteristics of the galls (e.g., organ appearance, size, color, and pubescence). For more details about sampling, please see Araújo et al. [12]. We used the host plant species, the galling insect morphospecies, and the interactions performed between them to build a plant–galling insect network for each study area.

### 4.3. Determination of Superhost Plants

**Figure 6:**
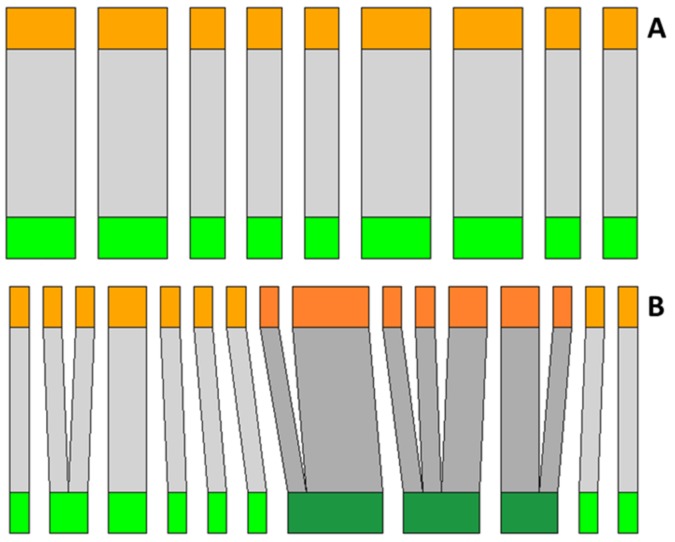
Examples of plant–galling insect networks sampled in the study. For each network, green bars represent host plant species and orange bars represent galling insect species. Gray bar thickness is proportional to the number of interactions of each species (drawn at different scales). (**A**) Structure of a network without the presence of superhost species (area 13; Table 2). (**B**) Structure of a network with presence of three species of superhost plants (area 5; Table 2). Species of *Qualea* and their galling insects are represented by dark green and orange bars, respectively.

Previous studies identified species of the genus *Qualea* (Vochysiaceae) as superhosts of galling insects in the Brazilian Cerrado [12,27]. The genus *Qualea* has wide distribution in the Cerrado, occurring in various types of phytophysiognomy, especially in the cerrado *sensu stricto* [26,28]. In our sampling areas, we recorded several species of galling insects on three species of *Qualea* (*Qualea grandiflora* Mart., *Qualea multiflora* Mart., and *Qualea parviflora* Mart.) (Table 2; Figure 6) [12]. In this sense, we used the occurrence of *Qualea* species (number of species) as an ordinal variable of occurrence of superhost plants to each sampled area that ranged from 0 to three species.

### 4.4. Data Analyses

In order to describe the structure of plant–galling insect networks, we used the following network descriptors: linkage density of plant species, number of observed interactions, network size, and network connectance [22]. Linkage density is the mean number of links per species of host plants, number of interactions is the number of associations between pairs of plant species and insects in the network (i.e., richness of interactions), while the network size was obtained from the sum of the number of plant species and insect species in the network (i.e., richness of interacting species). Network connectance is an inverse measure of overall interaction specialization [24]; thus, high connectance means low specialization in plant–galling insect networks. We calculated the connectance dividing the number of observed interactions by number of possible interactions (number of plant species multiplied by number of galling species in the network) to each network [22].

Due to the negative relationship between network size and connectance [32], we also used the residual connectance calculated as the residuals from a linear regression between the number of realized interactions and the number of potential interactions (both log-transformed) across plant–galling insect networks. This approach controls for the effects of network size and allows comparisons of connectance among different types of ecological networks [24]. We calculated all network descriptors using the *bipartite* package [35] from R program [36].

We tested the relationships between the occurrence of superhost plants and the structure of plant–galling insect networks (linkage density of plant species, number of observed interactions, network size, network connectance, and residual connectance) using generalized linear models (GLMs). In order to better exploit the results, we also performed additional analyses to test the effect of network size on the network connectance, and the effect of occurrence of superhost plants on the richness of host plant species. All models were submitted to a residual analysis to determine the adequacy of error distribution. All statistical analyses were performed in R environment [36].

## 5. Conclusions

Our results have shown that superhost plants alter the structure of plant–galling insect networks in Neotropical savannas. We found that occurrence of *Qualea* species increased the linkage density of plant species and the number of observed interactions due to the addition of more links in the networks, corroborating our hypothesis. However, contrary to expectations, the occurrence of superhost plants negatively affects network connectance, probably due to the effect that superhosts have on increasing network size. Recent studies have advanced the understanding of how floristic aspects influence the structure of ecological networks [37,38,39]. Among these studies, there is evidence that plant diversity may increase the specialization [38] or generality [37] of interaction networks involving insect herbivores and plants. Our study represents a step forward in relation to these previous studies, showing that superhost plants alter the structure of plant–herbivore networks, even without having a significant effect on plant diversity. Our results constitute the first empirical evidence showing the existence of superhost plant effects for the structuring of plant–insect networks and also highlight the importance of these hosts for the maintenance of diversity of species and interactions involving galling insects in Neotropical savannas.

## Figures and Tables

**Table 1:** Generalized linear models showing the effects of occurrence of superhost plants on the network structure of plant–galling insect networks in Brazilian Cerrado.

Network Measure	Deviance Resid.	Df	Resid. Dev	F-Value	P-Value
Linkage density of plant species	8.2298	11	8.1250	3.714	0.045
Number of observed interactions	351.56	13	626.04	7.300	0.018
Network size	784.00	13	1584.9	6.430	0.025
Network connectance	0.0050	13	0.0072	9.090	0.009

**Table 2:** Location of Neotropical savanna areas (Brazilian Cerrado) sampled in the study and occurrence of superhost plant species (*Qualea* species). Areas correspond to the numbers indicated in the Figure 5.

Area	Locality	Coordinates	Occurrence of Superhost Plants
1	Lajeado, GO	17°53′ S, 51°38′ W	*Q. grandiflora* and *Q. multiflora*
2	Banana Menina, GO	16°59′ S, 49°14′ W	*Q. grandiflora* and *Q. multiflora*
3	Senador Canedo, GO	16°43′ S, 49°06′ W	*Q. grandiflora*
4	Itanhangá, GO	16°33′ S, 49°17′ W	*Q. grandiflora* and *Q. parviflora*
5	Bela Vista, GO	15°57′ S, 48°56′ W	*Q. grandiflora*, *Q. multiflora* and *Q. parviflora*
6	Bom Sucesso, GO	16°42′ S, 49°02′ W	*Q. grandiflora*, *Q. multiflora* and *Q. parviflora*
7	UEG, GO	16°22′ S, 48°56′ W	*Q. grandiflora*, *Q. multiflora* and *Q. parviflora*
8	Pedreira, GO	15°50′ S, 48°55′ W	*Q. grandiflora*, *Q. multiflora* and *Q. parviflora*
9	Fazenda Geraldo, GO	16°40′ S, 48°18′ W	*Q. grandiflora* and *Q. parviflora*
10	Caldas Novas, GO	17°42′ S, 48°38′ W	*Q. grandiflora*, *Q. multiflora* and *Q. parviflora*
11	Caça e Pesca, MG	19°00′ S, 48°18′ W	*Q. grandiflora*, *Q. multiflora* and *Q. parviflora*
12	Floresta do Lobo, MG	19°05′ S, 48°09′ W	*Q. grandiflora* and *Q. multiflora*
13	APA Cafuringa, DF	15°31′ S, 47°57′ W	No *Qualea*
14	REBio Contagem, DF	15°37′ S, 47°52′ W	No *Qualea*
15	Porto Real, TO	11°00′ S, 47°13′ W	*Q. parviflora*

## Supplementary Materials

The following are available online at https://www.mdpi.com/2223-7747/8/10/369/s1, Table S1: Checklist of galling insect species and host plant species recorded in the study.
